# Where Do I Stand? Perceptions of Racialized Social Status Among Latine Immigrants

**DOI:** 10.1017/S1049096526101954

**Published:** 2026-03-23

**Authors:** Angie N. Ocampo-Roland

**Affiliations:** University of Pittsburgh, USA

## Abstract

This article examines Latine immigrants’ perceptions of group status relative to White and Black individuals, highlighting how these perceptions shape their understanding of the US racial hierarchy. Focusing on the role of social interactions, the analysis explores equitable interactions (e.g., with a neighbor, coworker, or friend) and nonequitable interactions (e.g., with a supervisor) and their association with perceived status relative to each group. It also considers how experiences of discrimination and anti-Latino treatment influence these perceptions. Findings indicate that respondents view Whites as more advantaged, whereas perceptions of Black Americans’ status remain ambivalent. Equitable interactions improve Latines’ perceived status relative to Whites but do not consistently improve their sense of status relative to Black individuals. Conversely, discrimination substantially diminishes perceived status relative to Whites and, to a lesser extent, relative to Black individuals. Although results suggest modest opportunities for coalition building with Black Americans, the findings indicate these alliances may be limited. Similarly, discrimination is a major barrier to Latine–White relations, leaving Latine immigrants feeling marginalized relative to both groups.

In their landmark book, McClain and Stewart (1995) show that intergroup competition and coalition politics are central dilemmas in the study of racial and ethnic politics. At the center of these two potential outcomes is the question of “power contests,” which highlights the importance of group “status differences” in shaping intergroup relations (126). Even though the scholarship on intergroup relations emphasizes perceived threat and cooperation, I argue instead for the importance of people’s *relative sense of status* compared to other racial groups and for better integrating this concept into the study of intergroup relations.

As relatively new entrants into the US racial hierarchy, Latine immigrants offer a compelling case for a number of reasons. First, they are coexisting with individuals whom they may not have encountered as much in the past. In some cases, the location where they arrive is more racially diverse than their origin context. At the same time, they bring with them Latin American racial ideologies (Hernández 2022; Roth and Kim 2013; Zamora 2022). Many also experience discrimination because of their ethnoracial group for the first time, as they enter a racial hierarchy that is organized differently.

As relatively new entrants into the US racial hierarchy, Latine immigrants offer a compelling case for a number of reasons.

Although Latines share discriminatory experiences with Black Americans, studies document conflict and competition between the two, as well as anti-Black attitudes among Latines (Jones-Correa 2011; McClain et al. 2006; Morin, Sanchez, and Barreto 2011; Ocampo and Flippen 2021; Ocampo-Roland 2025; Robertson and Roman 2024; Wilkinson 2014). Opportunities to build coalitions between the two groups are also fragile (Pérez, Robertson, and Vicuña 2022), and strategic incentives vary by context (Waters, Kasinitz, and Asad 2014). Although Latines generally view Whites positively, their views of Whites become more negative as they become more integrated into US society and recognize their marginalized position (Ocampo and Flippen 2021). Thus, I argue that exploring perceived group status can help us understand where Latines view their group relative to other groups.

This article presents the findings of an original 2007–8 survey conducted in Durham, North Carolina, then an emerging immigrant destination experiencing rapid growth of its Latine population. Durham is an interesting case study for several reasons. It was the site of the 2006 study by McClain and colleagues, “Racial Distancing in a Southern City,” a foundational work that shaped our understanding of Latine–Black relations after demographic change and laid the groundwork for more recent studies on Durham. In 1990, before the major influx of Latines into this city, Whites were the majority (52%), Black residents comprised 46% of the population, and Latines only 1.1% (McClain et al. 2006). By 2000, Latines comprised 8.6% of the population, and by 2009, this had increased to 12.2% (Flippen and Parrado 2012). The growth of the high-tech sector in the area encompassing Durham, known as the “Research Triangle,” created many lower-skilled service and construction jobs, attracting Latine immigrants from other areas in the United States.

Given Durham’s substantial share of both White and Black residents, the area provides a good opportunity to explore how Latines understand their position relative to both groups. The earliest Latine immigrants tended to settle in predominantly Black areas, but as the community grew, migrant neighborhoods that were more distant from Black neighborhoods gradually emerged (Flippen and Parrado 2012). Oral histories document the tensions spurred by this influx. Black residents of Durham showed empathy and acceptance toward Latines yet also resented the changes that Latines brought to their neighborhoods’ look and social atmosphere (Waugh 2024). Thus, the Durham case highlights the dynamics in a new immigrant destination in which an influx of Latine immigrants affects the overall social landscape.

The in-person survey, conducted via community-based participatory methods, captures a largely undocumented, recently arrived, and low-income population often missed by traditional surveys. It also includes unique measures of group status that are not available in nationally representative surveys. Additionally, the time period when the survey was conducted mirrors today’s immigration climate: it was marked by deportations, workplace raids, and xenophobic rhetoric sparking fear in the community (Collins 2007b). Although Durham local officials did not officially cooperate with federal immigration authorities to facilitate deportations (Arounnarath 2006), Senator Elizabeth Dole, who represented North Carolina then, pushed for increased enforcement. Dole “said she would seek more federal funding to help counties eager to participate in a program that allows them to enforce federal immigration laws” (Collins 2007a). She commented on the deportation program, saying, “Illegal aliens have committed crimes, often over and over and over again, and what can we do?” (Collins 2007a).

I draw on several measures to understand how Latine immigrants view their group status relative to both White and Black Americans. I expect that social interactions are relevant for shaping Latines’ perceived group status and that their impact depends on whether these interactions are equitable (neighbor or coworker) or nonequitable (supervisor). I also explore how discrimination shapes perceptions of status. Overall, results suggest that Latine immigrants perceive that Whites are relatively advantaged but are more ambivalent about their status relative to Black Americans. Equitable interactions do not always positively influence perceived status differentials, and nonequitable interactions do not always negatively influence these perceptions. The findings also reveal that discriminatory experiences are important in shaping perceptions of Whites and, to a lesser extent, Black Americans.

## RELATIVE SOCIAL STATUS AND THE STUDY OF RACIAL ATTITUDES

US society operates within a status-based racial hierarchy, yet measures of intergroup relations often focus on prejudice, conflict, or cooperation. Status reflects social superiority or inferiority (Chan and Goldthorpe 2007) and a “sense of being valued” (Ridgeway 2014), and it emerges through repeated interactions. These beliefs, rooted in unequal resource distribution, create durable associations between racial groups and relative status (Manago, Sell, and Goar 2022). Thus, I argue that examining perceived status *relative* to out-groups complements existing approaches by revealing how individuals position their group within the hierarchy. Adopting a relational lens allows us to consider factors beyond the perceived competition over finite resources and provides greater insight into perceptions of the racial hierarchy.

The racial hierarchy is relational; that is, groups’ positions relative to other groups shape status. Zou and Cheryan (2017) identify foreignness and inferiority as key axes, placing Whites at the top. Although Black Americans and Latines share the inferiority dimension, Black individuals are considered American, unlike Latines. Therefore, undocumented Latines are even more marginalized on this dimension. Furthermore, Latine immigrants view Black Americans as advantaged because of their citizenship (Zamora 2018). Thus, although Whites are at the top of the hierarchy, how the racial hierarchy structures Black–Latine relations is more complicated.

However, shared marginalization does not guarantee joint coalition building. For instance, the work on social dominance orientation suggests that a stronger desire to occupy a superior group position is associated with negative out-group attitudes (Pratto et al. 1994), even among lower-status groups like Latines (Levin et al. 2002; Sidanius and Pratto 2001). Indeed, many Latines employ distancing strategies from Black individuals (Cadena 2022) or other Latines (Hickel, Oskooii, and Collingwood 2024) to approximate whiteness (Ocampo, Ocampo-Roland, and Uribe 2025).

Although studies suggest Latines often hold anti-Black attitudes and view Whites more positively (Bobo and Hutchings 1996; Jones-Correa 2011; Krupnikov and Piston 2016; McClain et al. 2006), recent work highlights the importance of considering status, power, and relational dynamics. Wilkinson (2014) finds that economic threats reduce Latines’ sense of commonality with Black Americans. Conversely, discrimination against Latines, signaling lower status, can increase solidarity with Black individuals (Craig and Richeson 2012) and support for pro-Black policies (Pérez, Vicuña, and Ramos 2023), highlighting shared marginalization as a basis for coalition building. Solidarity with other people of color is also relatively stable among Latines (Engelhardt et al. 2025), yet is contingent on discrimination stemming from a shared status. On the contrary, when there are threats to Latines’ status as American, anti-Black attitudes increase, and support for pro-Black policies decreases (Pérez, Robertson, and Vicuña 2022). Furthermore, Zamora’s (2018) Los Angeles-based study captures how having negative attitudes may not reflect the complexities of a relational lens: Mexican immigrants see both Whites and Black Americans as superior because of their citizenship and report discrimination from both groups, suggesting barriers to coalition building despite shared struggles.

Understanding perceptions of group status can help us ascertain how racial groups view their positionality within the broader system. A mostly undocumented, recently arrived migrant population in Durham, amid xenophobic rhetoric and immigration raids, would likely view themselves as marginalized relative to White and Black individuals. These status perceptions could take several forms. Viewing White and Black Americans as relatively advantaged may signal recognition of the exploitation faced by undocumented Latines. Seeing Black individuals as less advantaged could reflect acknowledgment of their marginalization, fostering commonality and potential coalition building between the two groups (Craig and Richeson 2012; Pérez, Vicuña, and Ramos 2023). Conversely, perceiving Black individuals as more advantaged may suggest that they are seen as less promising allies. Respondents will likely view Whites as advantaged overall; however, if Whites are seen as less advantaged, Latines may perceive themselves as closer to them (Hickel, Oskooii, and Collingwood 2024; Ocampo, Ocampo-Roland, and Uribe 2025). This perception, combined with viewing Black individuals as more advantaged, could indicate prejudicial thinking tied to racial resentment.

Although these evaluations reflect perceived group status, resentment of White and Black individuals can shape the mechanisms underlying them. Latines may see Whites as more advantaged because of their higher socioeconomic position. Perceptions of Black individuals, however, may stem from anti-Black prejudice. For example, Zamora’s study found that although Latine immigrants viewed Black Americans as better off, they also described them as lazy and failing to seize opportunities, while “Whites are often absolved of any responsibility” (Zamora 2018, 1906). Therefore, prejudicial attitudes can influence status perceptions.

## WHAT SHAPES PERCEPTIONS OF RELATIVE SOCIAL STATUS?

A central aspect of the socialization of Latine immigrants into the US racial hierarchy is the kind of interactions that they have with out-groups. Although Latine immigrants do not arrive in the United States with a “blank slate” when it comes to racial attitudes (Zamora 2018) and often bring with them anti-Black attitudes from Latin America (Hernández 2022), their social interactions will likely inform their perceptions of where they fit into the racial hierarchy. According to contact theory, social interactions based on mutual cooperation with out-groups foster improved intergroup relations (Allport 1954). Despite the centrality of pursuing common objectives to Allport’s theory, scholars seldom focus on the opportunities for working together when exploring different forms of contact, often suggesting that most contact produces improved out-group attitudes. Although some research suggests that Latines having White neighbors (Britton 2014) and friends (Wilkinson 2014) is associated with more positive perceptions of Whites, other studies find that Latine attitudes toward Whites worsen with greater contact (Ocampo and Flippen 2021). Studies also find that Latine attitudes toward Black Americans improve with more contact (McClain et al. 2006; Ocampo and Flippen 2021). At the same time, certain kinds of social contact, such as having Black coworkers or neighbors, are associated with higher perceived competition (Carey et al. 2016; Jones-Correa 2011; Morin, Sanchez, and Barreto 2011; Wilkinson 2014). Overall, we can also expect experiences of discrimination to also negatively affect perceived relative social status (Flippen and Parrado 2015; Walker, Webster, and Bianchi 2011).

I expect that when examining the factors that contribute to perceived group status, the type of social interactions (equitable vs. nonequitable) that individuals have with each group should affect their perceptions of group status. This is an important differentiation in the nature of social contact that better reflects Allport’s (1954) original conditions, rather than suggesting that contact is uniformly a positive phenomenon. Among mostly undocumented and low-income Latine immigrants, I explore whether Latines perceive other groups to be more advantaged. This can involve whether they perceive members of these groups as taking advantage of Latines or of having greater advantage in society overall. I expect that equitable contact will result in a lower likelihood of perceiving that each out-group is more advantaged than Latines, whereas nonequitable contact and experiencing discrimination will result in perceiving each group as more advantaged relative to Latines. These are my three hypotheses:

H1:
Equitable contact (friend, neighbor, or coworker) will result in a lower likelihood of perceiving each group as relatively advantaged.

H2:
Nonequitable contact (boss) will result in a higher likelihood of perceiving each group as relatively advantaged.

H3:
Discrimination (experiencing discrimination across various settings and anti-Latino treatment) will result in a higher likelihood of perceiving each group as relatively advantaged.

## DATA AND METHODS

This study uses the findings of the *Gender, Migration, and Health Among Hispanics* survey (Parrado, McQuiston, and Flippen 2005) conducted in the Durham, Chapel Hill, and Carrboro metropolitan area in 2007 and 2008.^1^ Through community-based participatory research (CBPR), community members were integrated into the survey’s planning and data collection efforts, ensuring community buy-in and improving response rates, data quality, and recruitment of vulnerable populations (Flippen and Parrado 2015). More details on the process are available in the supplementary appendix, section B. The surveys were conducted in person by trusted community members, most of whom were Latine. Although co-ethnic interviewers are ideal for questions about perceived group status relative to other racial groups, some self-censoring of respondents’ attitudes caused by a social desirability bias is possible (White and Laird 2020). The questions about group status referenced here are not available in nationally representative surveys. Another important advantage of this survey is that it captures members of a predominantly undocumented population, who are traditionally overlooked in surveys in the social sciences. The total sample size is *N* = 676, of whom roughly half were men and half women (Ocampo-Roland 2026).

The first set of dependent variables examine perceptions of economic exploitation, specifically asking whether respondents think that White or Black people benefit economically at the expense of Latines. The second set of variables capture perceptions of relative group power and whether respondents perceive that Black or White people have too much power. These questions build on previous work underscoring how power is an important component of race relations (Wilkinson 2014; Zamora 2018).

The first set of independent variables include characteristics of social interactions with out-group members. These are dummy variables characterizing whether respondents have contact with either group as friends, neighbors, coworkers, or supervisors. To capture discrimination, two additive indexes were created that capture discrimination by White versus Black individuals: they include a series of questions asking respondents whether they were ever discriminated against in various settings (such as at school or when trying to obtain a job)^2^ and whether the race of the discriminator was Black or White. Respondents were then asked whether people from that racial group discriminated against them a few times (coded as 1) or many times (coded as 2) in that specific setting.^3^ If they did not indicate the race of the discriminator, the responses were coded as 0 for that particular setting. This results in two separate indexes, one capturing experiences of discrimination by Whites and another capturing experiences of discrimination by Black individuals. A third related variable is an additive index of whether respondents experienced anti-Latino/immigrant treatment. It compiles two measures: whether respondents did not feel accepted because of their culture and whether they perceived they were treated poorly because of their English-language ability. Previous studies that explored discrimination among Latines also used indexes to capture the compound nature of many discriminatory experiences (Flippen and Parrado 2015).

The next set of independent variables measure socioeconomic status—household weekly income and years of education—and acculturation: a binary variable for whether respondents speak at least some English or not. A dummy variable captures whether respondents have lived at least five years in Durham. Country of origin is another dummy variable, capturing Mexican versus non-Mexican origin. Lastly, I include a variable that captures whether respondents fear deportation. Respondents were asked whether they worry about being deported if they visit a social services agency and whether they avoid the police due to fear of immigration authorities. If respondents answered yes to either question, they were coded as fearing deportation.

## RESULTS

Table 1 presents descriptive results for the variables included in the analysis. Respondents generally have low incomes and levels of education; most are newly arrived migrants, and 90% are undocumented. Thus, respondents generally occupy a lower, more marginalized position.

**Table 1:** Descriptive Statistics

Dependent variables	%
Whites benefit economically at expense of Latines	79.82
Whites have too much power	86.32
Blacks benefit economically at expense of Latinos	66.57
Blacks have too much power	40.47
**Social interactions**	
Discriminated against by Whites	0.89 (mean)
	0–13 (range)
Discriminated against by Blacks	1.01 (mean)
	0–9 (range)
Experienced anti-Latino/immigrant treatment	1.28 (mean)
	0–2 (range)
Has White neighbors	58.38
Has White friends	44.51
Has White coworkers	54.91
Has White boss	67.05
Has Black neighbors	84.25
Has Black friends	29.62
Has Black coworkers	48.41
Has Black boss	28.90
**Sociodemographic characteristics**	
Male	51.01
Mexican	65.56
Speaks at least some English	67.73
Lived in Durham at least 5 years	44.80
Fears deportation	66.76
Years of education (mean)	7.91
Total household weekly income (mean)	512.4

Respondents are more likely to view Whites in a superior position than Black Americans. Around 80% of respondents believe that Whites benefit economically at the expense of Latines, whereas 67% believe this to be the case for Black Americans. Furthermore, the majority of respondents think that Whites have too much power (86%). This is more than double the percentage of respondents who think that Black Americans have too much power (40%).

The social interactions showcase that even though respondents are more likely to say they have Black neighbors (84%) than White neighbors (58%), they are also more likely to indicate that they have White friends than Black friends (45% vs. 30%). Respondents are only slightly more likely to indicate having White coworkers (55%) than Black coworkers (48%) but are more than twice as likely to report having White supervisors (67%) as Black supervisors (29%). This suggests that they have more opportunities for equitable contact with their Black counterparts. The majority of respondents said that they have not been discriminated against by Whites, thus making the mean number of discriminatory experiences slightly under 1 (0.89). Respondents were more likely to say they have been discriminated against by Black individuals, with the mean being 1.01. It is also important to note that the range is wider when considering discriminatory experiences by Whites compared to Black individuals (0–13 vs. 0–9): thus, a few respondents were more likely to indicate more discrimination across various settings from Whites. Furthermore, most respondents reported experiencing anti-Latino/immigrant treatment: the mean is 1.28.

### Status Compared to White Americans

**Figure 1:**
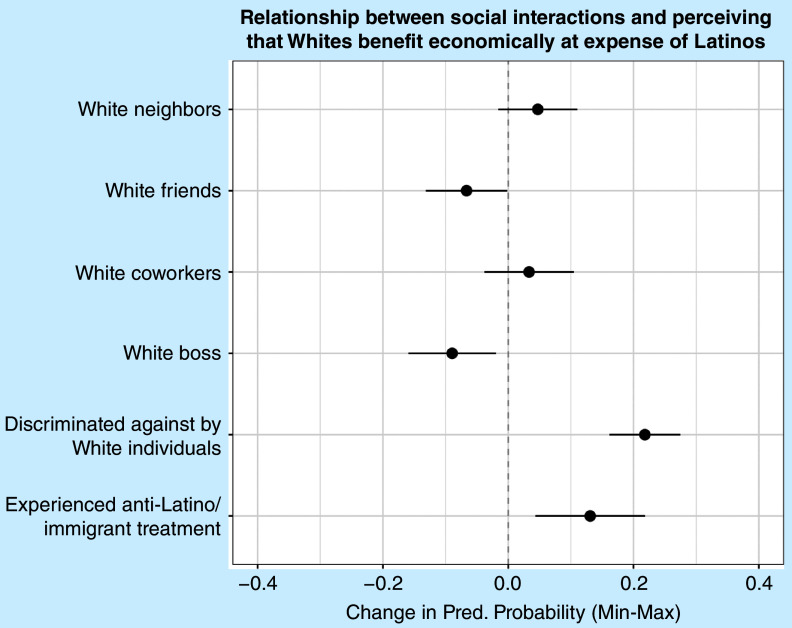
Predicted Probabilities from Logistic Regression Models Predicting Perceptions of Whether Whites Benefit Economically at the Expense of Latines

Figure 1 presents predicted probabilities from a logistic regression model of whether Latines perceive that Whites benefit economically at their expense (the corresponding logistic regression table can be found in supplementary appendix table A1, model 1). Considering the relationship between equitable social interactions and perceptions of Whites’ status, having White friends is associated with a 7% decrease in perceiving that Whites benefit economically at Latines’ expense. This provides support for H1. However, countering the expectation set out in the second hypothesis, having a White boss is associated with a 9% decrease in believing that Whites engage in economic exploitation of Latines. Even though having a White supervisor may not be an equitable interaction, it still results in improved perceptions of Whites. This finding is somewhat surprising, given that perceptions of economic exploitation are likely tied to the workplace.

Respondents who experienced negative treatment are more likely to perceive that Whites benefit economically at the group’s expense. Specifically, going from not being discriminated against at all by Whites to the highest amount of reported discrimination is associated with a 22% increase in the likelihood of believing that Whites benefit economically at Latines’ expense. Similarly, going from not experiencing anti-Latino treatment to experiencing such treatment at its highest level is associated with a 13% increase in the likelihood of believing that Whites engage in economic exploitation of Latines. These results support H3.

**Figure 2:**
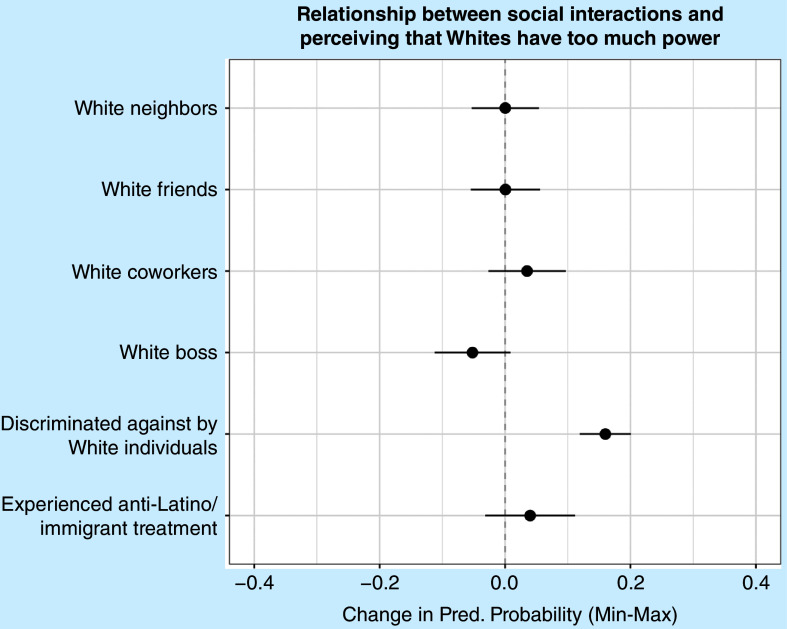
Predicted Probabilities from Logistic Regression Models Predicting Perceptions of Whether Whites Have Too Much Power

Figure 2 presents predicted probabilities from a logistic regression model of whether Latines perceive that Whites have too much power (corresponding to appendix table A1, model 2). Going from not reporting any discrimination perpetrated by Whites to reporting the highest level of discrimination by Whites results in a 16% increase in the likelihood of indicating that Whites have too much power. This supports the third hypothesis. However, the other social interaction variables do not significantly predict perceptions of power, which could be because a plurality of respondents think that Whites have too much power. At the same time, it is important to note the differences between the two questions that assess the perceived advantage of each racial group. Whereas the economic advantage variable asks whether respondents think that individuals engage in the direct exploitation of Latines, the power variable is more general and does not specify a dimension of power.

### Status Compared to Black Americans

**Figure 3:**
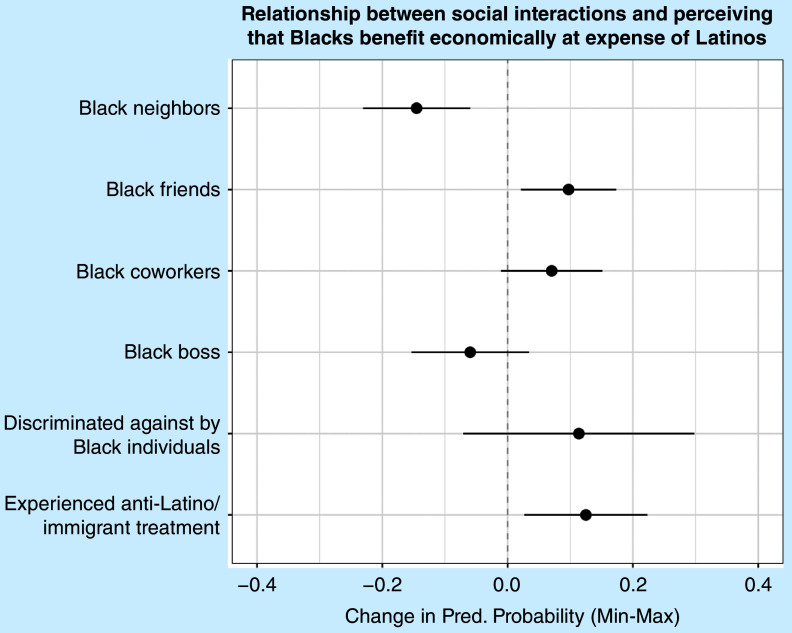
Predicted Probabilities from Logistic Regression Models Predicting Perceptions of Whether Blacks Benefit Economically at the Expense of Latines

**Figure 4:**
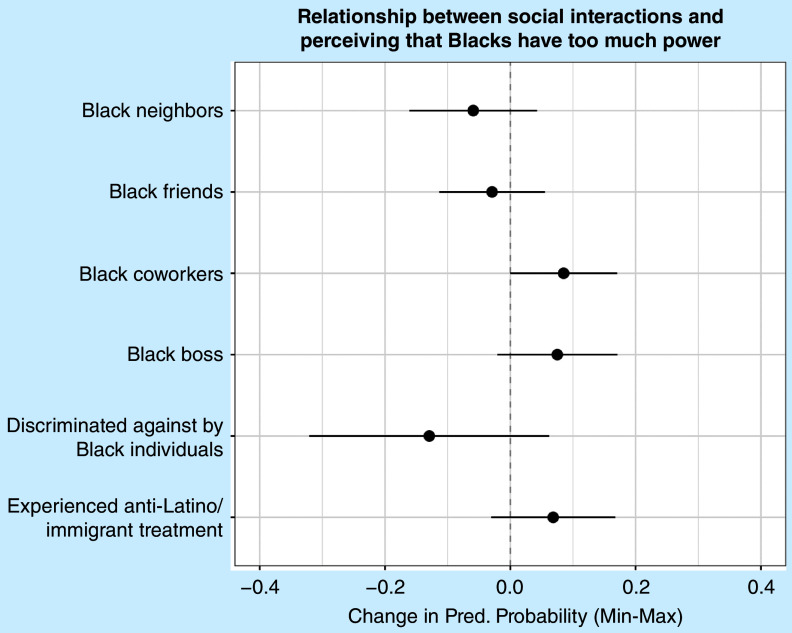
Predicted Probabilities from Logistic Regression Models Predicting Perceptions of Whether Blacks Have Too Much Power

Figure 3 presents predicted probabilities from a logistic regression model of whether Latines perceive that Black individuals take advantage of them economically (corresponding to appendix table A2, model 1). Having Black neighbors is associated with a 15% lower likelihood of perceiving that Black individuals benefit economically at Latines’ expense, which provides support for hypothesis 1. However, having Black friends is associated with a 10% greater likelihood of believing this to be the case, running counter to expectations set out in the first hypothesis. Having a Black boss does not significantly affect perceptions of economic exploitation: this finding does not support hypothesis 2. Although experiencing discrimination from Black individuals does not significantly predict perceptions of economic advantage, experiencing higher levels of anti-Latino treatment is associated with a 12% greater likelihood of believing so. Figure 4 presents predicted probabilities from a logistic regression model of whether Latines perceive that Black individuals have too much power (corresponding to table A2, model 2). However, social interactions and discrimination do not predict Latine perceptions of whether Black individuals have too much power.

## DISCUSSION

Using a survey of mostly undocumented Latine immigrants, this article enriches our understanding of how immigrants see their place in the overall racial hierarchy by exploring how social interactions affect perceptions of group status. Descriptively, Latines generally view Whites as more advantaged, and a smaller proportion believe Black individuals benefit economically at Latines’ expense. Most respondents, however, do not think that Black individuals hold excessive power.

Using a survey of mostly undocumented Latine immigrants, this article enriches our understanding of how immigrants see their place in the overall racial hierarchy by exploring how social interactions affect perceptions of group status.

Furthermore, the study explores how social interactions shape these perceptions. Interactions, even unequal ones, often improve Latines’ sense of status relative to Whites. For instance, having a White boss or friends lowers perceptions that Whites exploit Latines economically. Conversely, discrimination and anti-Latino treatment increase perceptions of White economic advantage. However, although having Black neighbors lessens perceptions of Black economic advantage, having Black friends is associated with perceiving that Black individuals are in a superior position. Experiencing anti-Latino treatment is also associated with a greater likelihood of perceiving that Black individuals are economically advantaged relative to Latines.

## CONCLUSION

This study offers insights into Latine immigrants’ perceptions of racial group status in a new immigrant destination, focusing on mostly undocumented, low-income individuals who are often excluded from surveys. Although it captures views of relative group status in terms of economic advantage and power, the underlying motivations for such perceptions, which can go beyond social interactions and discrimination, may also be intimately related to racial attitudes and racial positionality. Latines consistently see Whites as advantaged, although their views on Black individuals vary: some perceive them as relatively advantaged, whereas others do not. This contrasts with prior prejudice-focused research on racial attitudes, which finds that Latines view Black individuals as inferior (McClain et al. 2006). At the same time, it is also important to consider Blacks’ and Whites’ distinct positions in the racial hierarchy (Zou and Cheryan 2017) and what perceptions of status signify. Seeing Black individuals as higher status can coexist with anti-Black prejudice (Zamora 2018), whereas perceptions of White superiority may reflect their socioeconomic advantage or Latines’ aspirational whiteness (Ocampo, Ocampo-Roland, and Uribe 2025).

An important caveat is the difference between the two dependent variables. The economic advantage measure reflects perceptions of these groups benefiting economically at Latines’ expense, possibly indicating exploitation such as wage theft. The power measure, by contrast, captures perceptions of “too much” power, which may be interpreted more variably because the wording is less explicit. Another limitation is that these two measures only capture whether a group is viewed as superior and are less adept at capturing similarity. When considering the basis for multigroup coalitions, similarity based on shared marginalization is important (Pérez, Vicuña, and Ramos 2023).

A key takeaway is how different social interactions shape perceived status. Having White friends reflects an equitable relationship, whereas having a White boss involves a clear power imbalance. However, having a White boss may still meet some of Allport’s conditions of cooperation (Allport 1954) when workplace interactions are positive. Another explanation is that Latine immigrants were grateful for employment, especially at a time when local immigration crackdowns were “forcing employers to fire workers with invalid Social Security numbers” (Collins 2007b).

The link between social interactions and status perceptions of Black Americans does not fully align with expectations that equitable contact reduces status gaps. Surprisingly, having Black friends, seemingly the most voluntary interaction, is associated with perceiving that Black individuals exploit Latines economically. One explanation is that more distant ties, such as Black neighbors, foster more positive views. Respondents also appear hesitant to form close friendships with Black individuals, and friendship quality may matter. Prior research shows that distant intergroup friendships can be associated with negative attitudes (Munniksma et al. 2013), and friend networks beyond dyads can amplify prejudice if others in the network hold biased views (Turetsky and Shelton 2024). These findings suggest that similar contact affects attitudes differently across out-groups: the effect of White versus Black friendships diverges. This contrasts with McClain et al.’s (2006) Durham study, which found that contact reduced anti-Black prejudice. Future research should assess contact quality, rather than only focusing on whether contact occurs.

Another takeaway is that although discriminatory experiences influence perceptions of both groups, the magnitude of the effect of discrimination on attitudes toward Whites is striking. This effect outweighs the positive influence of social interactions, underscoring how harmful discrimination is for intergroup relations. These findings do not support research positing that Latines aspire to whiteness (Ocampo, Ocampo-Roland, and Uribe 2025), because discrimination powerfully reinforces Latine perceptions of marginalization. Furthermore, there are important takeaways for coalition building, because respondents who experience anti-Latino treatment view Black individuals as relatively more advantaged. Although discrimination can sometimes foster solidarity among marginalized groups, this finding aligns more with research showing that Latines express anti-Black attitudes when their Americanness feels threatened (Pérez, Robertson, and Vicuña 2022). Moreover, respondents report more discrimination from Black individuals than Whites, suggesting limits to coalition building based on shared discrimination (Corral 2020; Jones 2019; Pérez, Vicuña, and Ramos 2024; Robertson and Roman 2024). Prior work shows that discrimination strengthens in-group identity (Sanchez and Espinosa 2016), further constraining cross-group solidarity. In a context where Latine immigrants are highly marginalized, discrimination presents a strong barrier for cross-group solidarity.

Ultimately, Latine immigrants generally perceive Whites as occupying a dominant position in the racial hierarchy, whereas views of Black Americans are more ambivalent, suggesting limited but possible coalition-building opportunities with the latter group. Discrimination from Whites also overshadows any potential gains in perceived status that might arise from social interactions. The current anti-immigrant sentiments, mass deportations, and racial profiling raids, echoing the context in which the survey was conducted (Zepeda-Millán 2017), will continue to deepen feelings of marginalization among Latine immigrants. This effect will be pronounced for undocumented and low-income communities, which remain the primary targets of large-scale deportation and fear campaigns. At the same time, persistent barriers to Black–Latine coalitions leave many Latine immigrants feeling marginalized by both groups.

Ultimately, Latine immigrants generally perceive Whites as occupying a dominant position in the racial hierarchy, whereas their views of Black Americans are more ambivalent, suggesting limited but possible coalition-building opportunities with the latter group.

## Supporting information

10.1017/S1049096526101954.sm001 Ocampo-Roland supplementary material Ocampo-Roland supplementary material

## Data Availability

Research documentation and data that support the findings of this study are openly available at the Harvard Dataverse at https://doi.org/10.7910/DVN/DBUQSI.

## NOTES

1. 2007–8 was a unique period for race relations. Obama’s presidential campaign marked an era of racial progressivism. As immigration reform was one of Obama’s campaign promises (American Presidency Project 2012), Latines may have viewed Black Americans as more likely coalition partners. On the contrary, seeing a Black candidate campaigning for the nation’s highest office may have resulted in perceiving Black Americans as more advantaged.

2. See appendix section D for the questionnaire.

3. Alternative specifications testing these two variables as binary (whether respondents *ever* experienced discrimination by the racial group in question or anti-Latino treatment) are available on tables A3 and A4 in the appendix. Most substantive results remain the same.

## SUPPLEMENTARY MATERIAL

To view supplementary material for this article, please visit http://doi.org/10.1017/S1049096526101954.
