# Supplementary material for: Where Do I Stand? Perceptions of Racialized Social Status Among Latine Immigrants
Source: PS Polit Sci Polit. 2026 Mar 23:1–10. Online ahead of print. doi: 10.1017/S1049096526101954 (PMC13038853; doi:10.1017/S1049096526101954)
Supplement: Ocampo-Roland supplementary material [file S1049096526101954sup001.pdf]

Where Do I Stand? Perceptions of Racialized Social Status Among Latine Immigrants

Angie N. Ocampo-Roland

PS: Political Science and Politics
Appendix

A Tables and Figures

Table A1: Coefficients from Logistic Regression Models Predicting Perceived Social Status of White Individuals Among Latine Immigrants

	Dependent variable:	
	Econ Adv	Power
	(1)	(2)
Has White friends	−0.433*	0.006
	(0.214)	(0.250)
Has White neighbors	0.308	0.003
	(0.208)	(0.244)
Has White coworkers	0.219	0.308
	(0.238)	(0.273)
Has White boss	−0.627*	−0.487
	(0.268)	(0.305)
Experienced anti-Latino/ immigrant treatment (Index)	0.407**	0.174
	(0.133)	(0.154)
Discriminated against in any setting by White individuals (Index)	0.246**	0.300**
	(0.094)	(0.115)
Fears deportation	−0.060	0.413
	(0.223)	(0.249)
Mexican	−0.158	−0.706*
	(0.221)	(0.278)
Male	0.300	−0.016
	(0.219)	(0.253)
Lived in Durham 5 years or more	−0.0003	−0.055
	(0.216)	(0.251)
Years of education	−0.018	−0.061
	(0.032)	(0.039)
Total household income	0.001	−0.0002
	(0.0004)	(0.0005)
Speaks at least some English	−0.358	−0.402
	(0.256)	(0.304)
Constant	1.120*	2.731***
	(0.481)	(0.587)
Observations	675	676
Log Likelihood	-318.864	-253.484
Akaike Inf. Crit.	665.727	534.969
Note:		*p<0.05; **p<0.01; ***p<0.001

Table A2: Coefficients from Logistic Regression Models Predicting Perceived Social Status of Black Individuals Among Latine Immigrants

	Dependent variable:	
	Econ Adv	Power
	(1)	(2)
Has Black friends	0.491*	−0.130
	(0.205)	(0.193)
Has Black neighbors	−0.774**	−0.260
	(0.264)	(0.226)
Has Black coworkers	0.343	0.375
	(0.203)	(0.192)
Has Black boss	−0.287	0.327
	(0.228)	(0.210)
Experienced anti-Latino/ immigrant treatment (Index)	0.294*	0.153
	(0.115)	(0.113)
Discriminated against in any setting by Black individuals (Index)	0.066	−0.067
	(0.060)	(0.055)
Fears deportation	0.449*	0.191
	(0.187)	(0.187)
Mexican	−0.065	−0.168
	(0.187)	(0.178)
Male	−0.274	−0.600***
	(0.181)	(0.173)
Lived in Durham 5 years or more	0.003	−0.164
	(0.185)	(0.177)
Years of education	−0.060*	−0.007
	(0.028)	(0.026)
Total household income	−0.0004	−0.001**
	(0.0004)	(0.0004)
Speaks at least some English	−0.429	0.068
	(0.220)	(0.202)
Constant	1.567***	0.387
	(0.466)	(0.429)
Observations	672	673
Log Likelihood	-401.731	-431.674
Akaike Inf. Crit.	831.463	891.347
Note:	*p<0.05; **p<0.01; ***p<0.001	

Table A3: Coefficients from Logistic Regression Models Predicting Perceived Social Status of White Individuals Among Latine Immigrants (With Alternative Specifications)

	Dependent variable:	
	Econ Adv	Power
	(1)	(2)
Has White friends	-0.406	0.031
	(0.214)	(0.250)
Has White neighbors	0.308	0.015
	(0.208)	(0.243)
Has White coworkers	0.215	0.304
	(0.238)	(0.272)
Has White boss	-0.652^{*}	-0.499
	(0.271)	(0.307)
Experienced anti-Latino/ immigrant treatment (Binary)	0.841^{***}	0.383
	(0.234)	(0.270)
Ever discriminated against in any setting by White individuals (Binary)	0.616^{**}	0.596^{*}
	(0.230)	(0.262)
Fears deportation	-0.056	0.421
	(0.223)	(0.246)
Mexican	-0.165	-0.720^{**}
	(0.222)	(0.277)
Male	0.306	0.014
	(0.220)	(0.253)
Lived in Durham 5 years or more	-0.015	-0.053
	(0.216)	(0.250)
Years of education	-0.021	-0.060
	(0.032)	(0.038)
Total household income	0.001	-0.0001
	(0.0004)	(0.0005)
Speaks at least some English	-0.363	-0.386
	(0.257)	(0.304)
Constant	0.944	2.592^{***}
	(0.488)	(0.591)
Observations	675	676
Log Likelihood	-317.939	-255.087
Akaike Inf. Crit.	663.878	538.175
Note:	*p<0.05; **p<0.01; ***p<0.001	

Table A4: Coefficients from Logistic Regression Models Predicting Perceived Social Status of Black Individuals Among Latine Immigrants (With Alternative Specifications)

	Dependent variable:	
	Econ Adv	Power
	(1)	(2)
Has Black friends	0.479*	−0.129
	(0.204)	(0.193)
Has Black neighbors	−0.769**	−0.262
	(0.263)	(0.227)
Has Black coworkers	0.319	0.379*
	(0.203)	(0.192)
Has Black boss	−0.263	0.313
	(0.227)	(0.210)
Experienced anti-Latino/immigrant treatment (Binary)	0.481*	0.392
	(0.210)	(0.218)
Ever discriminated against in any setting by Black individuals (Binary)	0.236	−0.083
	(0.178)	(0.170)
Fears deportation	0.490**	0.170
	(0.185)	(0.185)
Mexican	−0.069	−0.162
	(0.187)	(0.178)
Male	−0.303	−0.592***
	(0.180)	(0.172)
Lived in Durham 5 years or more	−0.010	−0.180
	(0.185)	(0.177)
Years of education	−0.063*	−0.008
	(0.028)	(0.026)
Total household income	−0.0003	−0.001**
	(0.0004)	(0.0004)
Speaks at least some English	−0.435*	0.034
	(0.219)	(0.201)
Constant	1.528**	0.273
	(0.472)	(0.440)
Observations	672	673
Log Likelihood	-402.425	-431.361
Akaike Inf. Crit.	832.850	890.721
Note:	*p<0.05; **p<0.01; ***p<0.001	

B Sampling

Due to their highly marginalized position, Latine immigrants are difficult to sample in a locally representative manner (Brown 2015). Because the Latine community in Durham was recently settled at the time of the study, the researchers also employed targeted random sampling techniques, since simple random sampling would have been prohibitively expensive. After identifying 49 apartment complexes that housed a large number of Latine immigrants in the area, the researchers took a census of all of these apartments and randomly selected individual units for in-person surveys in Spanish. Although this technique may not capture more established immigrants in the area, it is preferable to using non-random methods such as convenience or snowball sampling. To address potential concerns of how the sampling procedure may have biased respondent selection, the researchers evaluated the sample in comparison to data from the 2000 Census and found no statistically significant differences on sociodemographic characteristics such as age, education, employment, wages, and time in the United States.

C Ethics

This project adheres to APSA's Principles and Guidance for Human Subjects Research.

D Questionnaire

Muchas gracias por colaborar con nuestro estudio. Vamos a empezar con preguntas sobre actitudes raciales.
Thank you very much for agreeing to participate in our study. We are going to start with questions on racial attitudes.

1. Por favor, dígame si a Usted le parece que...
   Please, let me know if you think that...

   - Los Am-blancos tienen demasiado poder?
     White Americans have too much power?
     Si / Yes
     No / No
   - Los Afro-Americanos tienen demasiado poder?
     African Americans have too much power?
     Si / Yes
     No / No
   - Los Afro-Americanos tratan de mejorar económicamente a costa de los Latinos?
     Do African Americans try to benefit economically at the expense of Latinos?
     Si / Yes
     No / No

- Los Am-blancos tratan de mejorar económicamente a costa de los Latinos?
  Do White Americans try to benefit economically at the expense of Latinos?
  Si / Yes
  No / No

Ahora le voy a hacer unas preguntas sobre como se ha sentido aqui en Durham/Carrboro.
Now I'm going to ask you some questions about how you have felt here in Durham/Carrboro.

2. Alguna vez ha experimentado discriminación, no le han permitido hacer algo, le han molestado o hecho sentir inferior en alguna de las siguientes situaciones debido a su raza, etnia, o color?
   Have you ever experienced discrimination, i.e. someone has not allowed you to do something, someone has bothered you or made you feel inferior in any of the following situations due to your race, ethnicity, or color?

   - En la escuela
     At school
     Si / Yes
     No / No
     - Cuál era la raza de las personas que le trataron mal, y cuántas veces le pasó?
       What was the race of the people who treated you poorly, and how many times did it happen?
       * Blanca / White
         Pocas veces / Few times
         Muchas veces / Many times
       * Afro-Americana / African American
         Pocas veces / Few times
         Muchas veces / Many times
   - Al obtener un empleo o en el trabajo
     When trying to find a job or at your job
     Si / Yes
     No / No
     - Cuál era la raza de las personas que le trataron mal, y cuántas veces le pasó?
       What was the race of the people who treated you poorly, and how many times did it happen?
       * Blanca / White
         Pocas veces / Few times
         Muchas veces / Many times
       * Afro-Americana / African American
         Pocas veces / Few times
         Muchas veces / Many times
   - Al rentar una casa
     When trying to rent a house

Si / Yes
No / No

- Cuál era la raza de las personas que le trataron mal, y cuántas veces le pasó? What was the race of the people who treated you poorly, and how many times did it happen?
    * Blanca / White
      Pocas veces / Few times
      Muchas veces / Many times
    * Afro-Americana / African American
      Pocas veces / Few times
      Muchas veces / Many times

- Al obtener asistencia médica
  When trying to get medical assistance
  Si / Yes
  No / No
  - Cuál era la raza de las personas que le trataron mal, y cuántas veces le pasó? What was the race of the people who treated you poorly, and how many times did it happen?
    * Blanca / White
      Pocas veces / Few times
      Muchas veces / Many times
    * Afro-Americana / African American
      Pocas veces / Few times
      Muchas veces / Many times
- Al pedir servicio en una tienda, restaurante o negocio
  At a store, restaurant or other business
  Si / Yes
  No / No
  - Cuál era la raza de las personas que le trataron mal, y cuántas veces le pasó? What was the race of the people who treated you poorly, and how many times did it happen?
    * Blanca / White
      Pocas veces / Few times
      Muchas veces / Many times
    * Afro-Americana / African American
      Pocas veces / Few times
      Muchas veces / Many times
- En la calle, en un lugar público
  On the street or at a public place
  Si / Yes
  No / No

- Cuál era la raza de las personas que le trataron mal, y cuántas veces le pasó?
  What was the race of the people who treated you poorly, and how many times did it happen?
  * Blanca / White
    Pocas veces / Few times
    Muchas veces / Many times
  * Afro-Americana / African American
    Pocas veces / Few times
    Muchas veces / Many times

- Con la policía o en las cortes
  With police or at the courthouse
  Si / Yes
  No / No
  - Cuál era la raza de las personas que le trataron mal, y cuántas veces le pasó?
    What was the race of the people who treated you poorly, and how many times did it happen?
    * Blanca / White
      Pocas veces / Few times
      Muchas veces / Many times
    * Afro-Americana / African American
      Pocas veces / Few times
      Muchas veces / Many times

Por favor, conteste si o no a las siguientes preguntas:
Please answer yes or no to the following questions:

3. Usted cree que las personas lo tratan mal porque usted no puede hablar Inglés bien?
   Do you think people treat you poorly because you can't speak English well?
   Si / Yes
   No / No

4. Usted siente que no es aceptado por otros debido a su cultura Hispana?
   Do you think that others do not accept you due to your Hispanic culture?
   Si / Yes
   No / No

5. Usted siente que será deportado si va a una agencia de gobierno o de servicio social?
   Do you think that you will be deported if you go to a government or social services agency?
   Si / Yes
   No / No

6. Usted evita la policía debido al temor de tener problemas con la migra?
   Do you avoid the police due to the fear of having issues with immigration?
   Si / Yes

No / No

Ahora, me gustaría hacerle unas preguntas sobre qué tanto se relaciona usted con diferentes tipos de personas.
Now, I would like to ask you some questions about how much contact you have with different kinds of people.

7. Pensando en los jefes o supervisores que tiene usted en el trabajo, tiene algunos que sean...
Thinking of the bosses or supervisors that you have at work, do you have any that are...
a. Am-blancos / White Americans
Si / Yes
No / No
b. Afro-Americanos / African Americans
Si / Yes
No / No

8. Y donde usted trabaja, sin incluir a los jefes, hay trabajdores que sean...
And where you work, without including supervisors, are there workers who are...
a. Am-blancos / White Americans
Si / Yes
No / No
b. Afro-Americanos / African Americans
Si / Yes
No / No

9. Pensando en las personas que viven en el vecindario, tiene vecinos que sean...
Thinking of the people that live in your neighborhood, do you have neighbors who are...
a. Am-blancos / White Americans
Si / Yes
No / No
b. Afro-Americanos / African Americans
Si / Yes
No / No

10. Pensando en sus amigos en Durham, tiene amigos que sean...
Thinking of your friends in Durham, do you have friends who are...
a. Am-blancos / White Americans
Si / Yes
No / No
b. Afro-Americanos / African Americans
Si / Yes
No / No

Ahora vamos de cambiar de tema un poquito. Voy a comenzar con unas preguntas sobre sus características generales.
Now we are going to change themes a bit. I am going to start with some questions about general characteristics.

11. Donde nació usted?
    Where were you born?

12. Cuántos años estudió usted?
    How many years did you attend school?

13. Qué tan bien habla usted Inglés?
    How well do you speak English?
    Muy bien / Very well
    Bien / Well
    Más o menos / Somewhat
    Para nada / Not at all

14. Cuánto tiempo ha vivido en Durham/Carrboro?
    How long have you lived in Durham/Carrboro?

15. Cuánto dinero gana en su trabajo principal?
    How much money do you make at your main job?

16. Es usted...
    Are you...
    Casado/a / Married
    En union libre / Civil union
    Soltero/a / Single
    Separado/a o divorciado/a / Separated or divorced
    Viudo/a / Widowed

17. (Si vive con pareja en EEUU) Cuánto gana su pareja en una semana típica?
    (If living with partner in U.S.) How much does your partner make in a typical week?
